# The post-pericardiotomy syndrome causing cardiac tamponade and pleural effusion in a patient that underwent mitral valve replacement

**DOI:** 10.1186/1749-8090-10-S1-A112

**Published:** 2015-12-16

**Authors:** Kazim Ergunes, Hasan Iner, Ismail Yurekli, Orhan Gokalp, Ufuk Yetkin, Ali Gurbuz

## Background/Introduction

The post-pericardiotomy syndrome (PPS) is an important complication following cardiac surgery.

## Aims/Objectives

We presented a patient with pericardial tamponade and pleural effusion that underwent mitral valve replacement.

## Method

A 45-year-old man was hospitalized in our clinic on March 26, 2015. He had chest pain, dyspnea, and easy fatigability. He underwent mitral valve replacement one month ago. The diagnosis of pericardial effusion was confirmed by echocardiogram. The chest X-ray showed left pleural effusion.

## Results

Subxiphoid pericardial drainage and right thoracic pleural drainage were performed under local anesthesia. Pericardial serous fluid of 400 ml was drained. The right pleural serous fluid of 500 ml was drained. A subxiphoid pericardial and right thoracic drainage tube were inserted during surgery and removed after 4 days. No microorganism was cultivated from pericardial and pleural fluids. After hospital discharge, patient was followed with physical examination, echocardiography, and chest X-ray.

## Discussion/Conclusion

Subxiphoid pericardial window under local anesthesia is an important surgical method for pericardial drainage in patients with pericardial effusion due to post-pericardiotomy syndrome.

## Consent

Written informed consent was obtained from the patient for publication of this abstract and any accompanying images. A copy of the written consent is available for review by the Editor of this journal.

